# Our Contemporaries

**Published:** 1914-09

**Authors:** 


### OUR CONTEMPORARIES

---

The Buffalo Times has recently voiced an objection, similar to that of the Evening Post of N. Y. (See page 225, August issue) against compulsory medical examinations at stated intervals, although acknowledging the advisability of such prophylactic measures.

---

Paediatrics suggests both federal and state legislation to control the sale of mercuric chlorid; limitation of the use of the drug to physicians for their personal use and to hospitals; allowing the sale to the laity of tablets containing approximately half a gram of bichlorid of mercury and of ammonium chlorid and also about 8 centigrams of tartar emetic, with the view to producing emesis if swallowed. It occurs to us that emetic action might occasionally occur from the injection of solutions into cavities.

---

The Laboratory News, formerly edited by Dr. R. B. H. Gradwohl of St. Louis has been merged with the Medical Fortnightly.

---

Florida has begun the publication of a state medical journal.

---

The Surgical Publishing Company of Chicago, publishers of Surgery, Gynaecology and Obstetrics with International Abstracts of Surgery announces their removal to 30 North Michigan Avenue, Chicago.

